# Hospital Utilization and Mortality Post-electrical Cardioversion in Patients With Atrial Fibrillation in a Community Hospital

**DOI:** 10.7759/cureus.66919

**Published:** 2024-08-15

**Authors:** Ashali Jain, Carolina Borz-Baba, Dorothy Wakefield

**Affiliations:** 1 Department of Medicine, Saint Mary's Hospital, Waterbury, USA; 2 Department of Statistics, Saint Francis Hospital & Medical Center, Hartford, USA

**Keywords:** hospitalized patients, atrial fibrillation (af), unsuccessful electrical cardioversion, 30-day readmission, mortality

## Abstract

Background

Electrical cardioversion (EC) is a procedure that restores normal sinus rhythm in patients with atrial fibrillation (AF). Data on post-EC outcomes relative to the success of inpatient EC is limited.

Methods

This is a retrospective study of patients admitted for AF who underwent inpatient EC from January 1, 2017, to January 1, 2021. We collected demographics and clinical, biochemical, and echocardiographic parameters that impact the success of EC. Outcome events were 30-day readmissions and mortality.

Results

Our study included 54 unique patients who either had EC in the emergency room or as part of their hospital admission course for atrial fibrillation. Most patients were men with an average age of 70 years with traditional risk factors for cardiovascular disease including heart failure, coronary artery disease, and chronic kidney disease. The group who had unsuccessful cardioversion was older than those in the ineffective EC. Mortality at 30 days (p < 0.01), 1 year (p < 0.01), and 30-day readmission rate (p < 0.01) were higher in patients with unsuccessful EC.

Conclusion

A predictive model for successful EC remains difficult to establish. Patients with unsuccessful in-hospital EC are at high risk for mortality and readmission at 30 days and require a comprehensive pre-discharge multidisciplinary approach and prioritized and individualized post-discharge integrated care.

## Introduction

The 2023 American Heart Association (AHA) Guidelines [1] state that patients with atrial fibrillation (AF) benefit from rhythm control to improve their symptoms, decrease the progression of AF, and reduce hospitalizations and mortality in patients with heart failure (HF).

Electrical cardioversion (EC) is recognized as a nonpharmacological treatment used to restore normal sinus rhythm (NSR) [1]. In hospitalized patients, acute rhythm control via EC is commonly employed in hemodynamically unstable patients or patients with preserved hemodynamics who either continue to be symptomatic from AF despite rate control or when rate control is suboptimal.

There is an increasing trend in hospital-based EC from 4.26% (between 2000 and 2010) [2] to 16.4% in 2014 [3]. Considering the rapidly escalating use of EC, a deep understanding of the current implementation of AF guidelines and resource utilization post-EC in hospitalized patients at community hospitals is imperative. Our study had two main objectives. The first goal was to investigate clinically predictive prototypes of successful versus unsuccessful EC. The second objective was to assess healthcare utilization and mortality in hospitalized patients who underwent EC.

## Materials and methods

Design

Investigators conducted a retrospective cohort study at a community teaching hospital in central Connecticut. We identified patients diagnosed with AF who underwent emergent or scheduled inpatient EC from January 1, 2017, to January 1, 2021. A successful EC was defined as post-procedure restoration and maintenance of NSR throughout the hospitalization. At our hospital, EC is performed by emergency room physicians, hospitalists, and cardiologists certified to use optimal techniques. A transesophageal echocardiography was performed before a non-emergent procedure. The study was approved by the Trinity Health of New England Institutional Review Board.

We excluded patients who had AF after percutaneous coronary intervention (PCI), patients initially admitted with CVA or intracranial hemorrhage, patients who returned to NSR with medical management, patients in whom cardiology specifically recommended only rate control, and those who left against medical advice.

Patient characteristics

We included all patients between the ages of 18 and 89. The information collected included age and gender, cardiovascular comorbidities including a history of HF, coronary artery disease (CAD), renal failure, and a prior history of AF. Objective findings of HF included the ejection fraction obtained via echocardiography completed during admission. To further appreciate the impact of HF with reduced EF (HFrEF) on the success of EC, we separately analyzed patients with an EF <40%.

Outcomes

The 30-day readmission and mortality at one year were obtained via chart review.

Statistical analysis

Descriptive statistics were used for all clinical and demographic variables. Clinical and demographic variables were compared for the two groups: EC successful versus unsuccessful. Chi-square analyses were used for categorical variables. Fisher’s exact tests were used when cell size was <5. For age, we compared groups using t-tests. The software utilized for statistical analysis was SAS 9.4 (SAS Institute, Cary, USA). A p-value of <0.05 was considered statistically significant.

## Results

Patient characteristics

In total, 54 patients underwent EC in the emergency department or as part of their hospital admission course for AF. Patient baseline demographic and clinical characteristics are provided in Table 1. More than half of the sample was comprised of men (57%). Approximately one-third (31.5%) had an ejection fraction (EF) of less than 40%, HF (63%), CAD (24.1%), prior history of AF (31.5%), and a GFR of less than 60 ml/min (33.3%). Measured baseline demographic and clinical characteristics of the population are provided in Table 1.

**Table 1:** Baseline characteristics of patients EF: ejection fraction; GFR: glomerular filtration rate

Patient characteristics	All patients (n = 54)
Age, years, mean (SD)	70.6 (11.1)
Sex, n (%)	
Male	31 (57.4)
Female	23 (42.6)
Prior history of AF, n (%)	17 (31.5)
EF <40%, n (%)	17 (31.5)
Heart failure, n (%)	34 (63.0)
Coronary artery disease, n (%)	13 (24.1)
GFR <60 ml/min, n (%)	18 (33.3)

Successful versus unsuccessful cardioversion

Of the cohort, nine patients (16.67%) had unsuccessful EC. Patient comorbidities and demographics were compared by the successfulness of cardioversion (Table 2). No statistically significant differences were found among the subgroups, though there was a near-significant difference in age. Patients who had unsuccessful cardioversion tended to be older than those with successful cardioversion (76 vs. 69 years; p = 0.09). Of the 45 patients who had a successful EC, 18 (40%) had a recurrence of AF.

**Table 2:** Patient characteristics by cardioversion successfulness EF: ejection fraction; HF: heart failure; CAD: coronary artery disease; GFR: glomerular filtration rate

Patient characteristic	Successful (n = 45)	Unsuccessful (n = 9)	p-value
Age years, mean (SD)	69.4 (11.4)	76.33 (11.6)	0.09
Sex (male), n (%)	26 (57.8)	5 (55.6)	1.00
EF <40%, n (%)	14 (31.1)	3 (33.3)	1.00
HF, n (%)	27 (60)	7 (77.8)	0.46
CAD, n (%)	10 (22.2)	3 (33.3)	0.67
Prior history of AF, n (%)	13 (28.9)	4 (44.4)	0.44
GFR <60ml/min, n (%)	14 (31.1)	4 (44.4)	0.46

Readmission at 30 days and mortality

Within 30 days of discharge, among the nine patients with an unsuccessful EC, three patients died and five of the other six were readmitted to the hospital. In contrast, only 4 of 45 (8.9%) patients with successful EC were readmitted within 30 days. Of the nine patients readmitted within one month of hospital discharge, 55.6% had a recurrence of AF. The 30-day readmission rate, as well as the 30-day and 1-year mortality rates, were all significantly higher in patients with EC failure than in patients with successful EC (Table 3).

**Table 3:** Readmission and mortality rates

Patient characteristic	Successful (n = 45)	Unsuccessful (n = 9)	p-value
30-day readmission, n (%)^1^	4 (8.9)	5 (83.3)	<0.01
30-day mortality, n (%)	0 (0)	3 (33.3)	<0.01
1-year mortality, n (%)	3 (6.7)	5 (55.6)	<0.01
^1 ^Excludes 3 patients in the unsuccessful EC group who died within 30 days			

## Discussion

Until 2023, the AHA guidance regarding EC in AF was restricted to patients who did not respond to pharmacological therapies and patients with hemodynamic instability or pre-excitation syndrome. The 2023 AHA [1] Guidelines provided specific and expanded recommendations regarding EC as a rhythm control strategy in AF.

The current study included patients who underwent inpatient EC from 2017 to 2021, before the updated AHA guidelines. Although not included in the study aims, we observed that in 2021, the incidence of EC in hospitalized patients with AF was 30% at our community hospital. This result indicates an incidence 10 times higher than that reported by Rochlani et al. [2] and almost double the most recent one published in 2014 [3]. These observations denote two important points. One is that common practice in community hospitals anticipated the need for new guidelines. The other reality is that the prevalence of ECs performed is considerably underestimated and calls for contemporary evidence.

Successful versus unsuccessful EC prognostic parameters

The EC success rate in our study was 83.3%. According to prior studies [4] and consistent with our results, effective EC is reported in 50-90% of cases, making it an appealing option for rhythm control with immediate effect and rare adverse events [4]. The success of EC depends on the technique [4], clinical factors, and biochemical and echocardiographic parameters.

In comparing clinical predictors of successful EC versus EC failure, we identified that patients with ineffective EC were older (p = 0.09). Although borderline statistically significant, the result is similar to findings reported in other recent studies [5-6]. The association between AF and advanced age is well-recognized [3]. The correlation between increasing age and ineffective EC is also expected as structural remodeling of the atrial myocardium and age-related predominance of comorbidities contribute to unsuccessful procedures. Yet, age alone cannot be a reason to defer EC.

Besides advanced age, various clinical factors that prevent successful cardioversion are male gender, a higher BMI [7], the presence of CAD [7-11] or HF [12,13], renal failure [5], a history of AF within 30 days before EC, and a permanent pacemaker [12]. More current evidence identified that a history of AF (>5 years) and a duration of index AF of >30 days [5] are also among prognostic factors. Except for the advanced age, our study did not replicate the statistical significance of the aforementioned unfavorable clinical criteria, likely due to a small sample size.

It is worth mentioning the role of biomarkers as predictors in AF. Clear and ample evidence demonstrates a correlation between inflammation and AF [14] and a variety of biomarkers are associated with the occurrence of AF [15]. A biomarker-related approach [15] is now promoted as an integrated part of multi-model strategies to anticipate the recurrence of AF, specifically after EC.

Echocardiographic parameters, especially an EF <40 and an increased cardiac size [12-13] are prognostic of ineffective EC. Our study did not collect specific echocardiographic features, including left atrial size, volume, left atrial appendage-flow velocity, the presence of pulmonary HTN, etc.; details not commonly available in patients who receive EC in the ED. Although frequently discussed, their usage in selecting responders to EC among hospitalized patients with a rapid ventricular response is not uniformly applied [16].

Although tempting, selecting specific clinical, biochemical, or echocardiographic characteristics of potential non-responders is challenging. Given the low to moderate evidence of the studies and the diversity of potential influencers, creating an algorithm or a prognostic model for effective EC, although beneficial, is premature.

Readmission at 30 days and mortality

According to the most recent report from the Nationwide Readmission Database [17], patients priorly hospitalized for AF have a rate of 30-day readmission of 14% with AF being the main cause of rehospitalization. In that study, 8.5% of patients had EC during index hospitalization and that was associated with a decreased readmission rate and cost of care [17]; however, it is unclear if all patients who underwent EC were discharged in NSR.

As expected, prior readmission and mortality correlate, with unplanned 30-day readmission being associated with a two-fold higher adjusted risk of death [16]. In newly diagnosed non-valvular AF mortality event rates at two years are lower in patients who had EC (2.52) compared with patients who did not have the procedure (3.87) [18]. Hence, supporting evidence favors in-patient EC vis-a-vis all-cause mortality among patients hospitalized for AF [2].

This study demonstrated that 16.6% of patients who had EC were readmitted at 30 days and more than half presented with a recurrence of AF. Readmission rate, mortality at 30 days, and 1 year were each significantly statistically (p < 0.01) higher for those who had unsuccessful cardioversion during the index hospitalization.

To our knowledge, our study is the first to report hard outcomes (readmission rate and mortality) in patients who had unsuccessful cardioversion. There are probably multiple reasons for the lack of evidence in this population. One is that research is commonly directed to acquire knowledge about prognostic factors of a successful procedure or treatment. The others are related to the complexity of such patients including their advanced age, frailty, and multiple acute or chronic comorbidities, variables difficult to control for in a study.

The cost of care in patients who are readmitted for AF is on the rise [19]. To avoid AF-related hospitalizations [20] or to reduce mortality [21] the evidence supports integrated care via a multidisciplinary approach. Integrated care includes a detailed evaluation of patients, coordination of care, patient education, and execution of plan [20]. Defining a comprehensive assessment of patients is challenging and includes clinical parameters and social determinants of health. Patients with AF and a high risk for mortality have vast benefits from such programs [22]. To our knowledge, the contemporary integrated care models do not include completions of procedures and their effectiveness (EC, electrophysiology studies) in their roadmaps. We propose here that the type of interventions and their successfulness should be incorporated into the clinical assessment of patients with AF before their discharge and in community-based integrated care programs. System-based interventions focusing on patient-centered transition of care for patients who failed EC, e.g., digital health, expedited evaluation by electrophysiology in the outpatient setting could reduce healthcare dollars and decrease mortality in AF.

Study limitations

Our study was single-center, community-based retrospective research. The study sample size was small, preventing the investigators from defining clinical prognostic factors for a successful versus unsuccessful EC. Despite a small cohort size, the correlation between the procedure outcome and critical clinical outcomes is supported by statistically significant (p < 0.01) results that leave little room for doubt of their value.

## Conclusions

The recent expansion of indications for EC predicts a significant increase in hospital utilization of EC, particularly in community-based hospitals. Our study demonstrates that understanding and exploring the link between unsuccessful cardioversion and outcomes is crucial for patients with AF and points toward the need for larger studies or subgroup analyses of already established studies with the scope to investigate clinical, biochemical, and echography predictors of EC failures. The increase in readmission and mortality at 30 days in this population prompts us to advocate for including outcomes of procedures in the AF hospital pathways and community-based integrated care for an individualized approach.
